# A dynamic career in MS: applications to biomedical research

**DOI:** 10.4155/fso.15.52

**Published:** 2015-11-01

**Authors:** Ian A Blair

**Affiliations:** 1 Penn SRP Center & Center for Excellence in Environmental Toxicology, Department of Systems Pharmacology & Translational Therapeutics, 854 BRB II/III, 421 Curie Blvd, University of Pennsylvania, PA 19104, USA

## Abstract

**Ian A Blair speaks to Francesca Lake, Managing Editor**: Blair received his PhD in Organic Chemistry in 1971 from Imperial College of Science and Technology, London, under the mentorship of the 1969 Nobel Laureate, Sir Derek HR Barton. He was appointed as the AN Richards Professor of Pharmacology at University of Pennsylvania in 1997 and Director of a new Center for Cancer Pharmacology. In 2002, Blair was appointed as Vice-Chair of the Department of Systems Pharmacology and Translational Therapeutics. In 2014, he became Director of the NIEHS-funded Penn Superfund Research and Training Program Center. Blair is an expert in the use of mass spectrometric methods for the structural elucidation and quantification of endogenous biomolecules. His current research is involved with the development of biomarkers in order to establish genetic/phenotype correlations and to assess the interaction between genes and exposure to environmental chemicals. He is particularly interested in the regulation of cellular oxidative stress and how this underpins mechanisms involved in carcinogenesis, cardiovascular disease and neurodegeneration. Blair discovered electron capture atmospheric pressure chemical ionization, a technique that makes it possible to conduct high sensitivity quantitative analyses of chiral biomolecules. He is a Fellow of the American Association for the Advancement of Science and the American Association of Pharmaceutical Scientists. He received the 2011 Eastern Analytical Award for Outstanding Achievements in Mass Spectrometry. Blair is Senior Editor of *Future Science OA*, and a member of the Editorial Board of several journals.

## Q What led you to choose research in pharmacology?

I was a research fellow in Adelaide, South Australia where I was working on steroid production rates as a trained organic chemist. I was making heavy isotope-labeled molecules to give to people to then see what happened. The way we analyzed what happened to the labeled steroids was by using GC–MS, so I got very interested in the technique of MS; it was more satisfying than the synthesis. I had just come from a postdoctoral fellowship where I was making gibberellic acid, which has eight chiral centers and is extraordinarily difficult synthetic target. Then one day I found out you could buy it for a dollar a gram and I wondered why on Earth I was doing all of this convoluted synthesis! My new work was much more fun because you gave the labeled steroids to people and they metabolized them at different rates; that was my entrée into pharmacology.

After that, I saw a job advertised in *Science* at the Royal Postgraduate Medical School at London University, which is now part of Imperial College (London, UK) and where I graduated. I applied for the job, and before I knew it, I was back in London after having been away for 9 years. I was a lecturer in Clinical Pharmacology, yet my knowledge of pharmacology was limited to what I had been doing with steroids. I spent 4 very productive years there learning all about pharmacology and developing many new MS-based assays. As a result of my studies, I was offered a position at Vanderbilt University in Nashville (TN, USA). I went there as a Professor of Pharmacology, going from clinical to regular pharmacology, but I also had an appointment in the Division of Clinical Pharmacology, so I was able to continued my translational research. I worked at Vanderbilt University for 13 years before I was recruited to the University of Pennsylvania (PA, USA) by Garret FitzGerald in 1997 to start a new Center for Cancer Pharmacology, based on studies I had performed on DNA adducts, DNA damage and genotoxicity. I kept these themes going for a number of years until more recently, when I have become more interested in mitochondrial dysfunction. My laboratory has now almost completely switched from analyzing DNA damage to looking at mitochondrial dysfunction and oxidative stress.

## Q What exactly are you working on at the moment?

Oxidative stress provides one of the major underlying mechanisms for causing DNA damage. For many years this was a major them of my research program. As part of this research, we discovered many new DNA adducts and new mechanisms of genotoxicity. Because of that interest, I was asked to lead a big program on asbestos, which is a compound thought to cause mesothelioma through a pathway involving oxidative stress. The vehicle for funding this research was through the National Institute of Environmental Health Sciences, who have a program called the Superfund Research and Training Program. We competed here at Penn – myself and 14 other people – to get one of these research and training programs and by some miracle we were successful. One of the reasons we were successful is because 10 years ago, Trevor Penning founded a Center of Excellence in Environmental Toxicology, which included a Translational Biomarker Core. I am the Director of that Core, which by working closely with Clementina Mesaros developed many of the techniques that turned out to be very useful in the Superfund proposal. Apparently, we were only the second Superfund Research and Training Program Center to be funded on its first submission. I calculated that we had <1 % chance of getting funded!

Superfund research is now one of the major themes of my laboratory. It is an interesting way of working as we attempt to address many of the issues and concerns of the community living close to the superfund site in Ambler, PA, USA. I meet regularly with the Community Advisory Group and describe the progress we have been making and listen to their concerns about what we can do in the future. The program is really starting to take off now. My main project in the center is to discover biomarkers for mesothelioma from a perspective that if we could identify people at risk for mesothelioma early, we could treat them early and there would be an increased chance of survival. Another component of my project is to develop biomarkers of asbestos exposure itself before any progression to mesothelioma. I think where this is going to be really useful is for people who live near the site, to know that they have not been exposed to asbestos. That component of my project is looking exciting now as we have discovered one biomarker of asbestos exposure that we are about to validate.

This theme of oxidative stress has also moved me in a totally different direction: a genetic disease called Friedreich's ataxia, which is consuming a lot of my attention. The disease is caused by a triplet GAA repeat in intron 1 of the frataxingene. This causes silencing of the gene involved in the expression of a protein called frataxin. This means that patients with the disease are unable to express frataxin, a protein that is involved in iron sulfur cluster assembly and mitochondrial metabolism. Over the last year, we have developed some interesting ways of monitoring whether the treatments that are being used for Friedreich's ataxia are having any effect on mitochondrial function. We use platelets from the Friedreich's ataxia patients and examine how they metabolize different precursors such as glucose, palmitic acid and isoleucine. We are now working with a drug company to examine whether their drug is having any effects on this pathway in patients. Thirty years of working on oxidative stress has led me into these two quite different areas: one Friedreich's ataxia and one asbestos exposure.

## Q Which of these areas appeals the most to you?

It is really difficult to decide. It is humbling to meet individuals in the Ambler, PA site on the one hand, and then giving presentations to patients with Friedreich's ataxia is also very humbling, too. Patients with Friedreich's ataxia tend to be in wheelchairs and their speech is affected so it is often hard for them to talk to you. Sadly, there are no treatments for this devastating disease. This places a tremendous responsibility on us researchers to come up with a treatment. These issues have stimulated me (as a scientist who normally spends all of his time in the laboratory) to become very involved with the Ambler community who are affected on the one hand, and with the patients with Friedreich's ataxia on the other hand. I feel that someone like me, with 30 years of experience in the area of oxidative stress, should be able to come up with a way of treating the terrible disease of Friedreich's ataxia and, in the case of asbestos exposure, finding a way of early detection so that we can treat people earlier. I would not like to say that one is more important than the other. Seeing a 6-year-old in a wheelchair pulls at your heartstrings, but then on the other hand, talking to someone whose brother had died from mesothelioma does that too. I feel the responsibility to use the resources that I have available to me to come up with solutions to these two issues.

## Q Do you find that working so closely with the people affected has an effect on your research more so than if you were solely in the laboratory?

Absolutely. I think it is not just about the patients themselves; it is the tough decisions that their parents have to make. This can be a nightmare. Spending time with the parents of affected with Friedreich's ataxia provides some insight into what the have to deal with. For example, if one child in the family is affected it is possible that another child in the family could be affected too, because there is a late-onset form of the disease. Parents are faced with all sorts of other difficult decisions such as how do you tell a child that he or she is affected, and then do you test the other children to see if they carry the triplet repeat or even a mutation. About 2% of Friedreich's ataxia patients have a mutation in the frataxingene in addition to the triplet expansion. I was recently at the International Ataxia Research Conference held 25–28 March 2015 (Old Windsor, UK), which had a whole session on how parents deal with these issues. It was fascinating but heartbreaking to think parents have to face those choices. To answer your question, it absolutely does affect the way I research.

## Q How do you personally deal with all of that?

I am fortunate that I do not have to make those choices; in fact I do not know what I would do myself. The average age of death is 37 years and the progression is slow. Most Friedreich's Ataxia patients become increasingly debilitated as they grow older and they eventually die of heart failure. I have to say that the people who work on this disease here at Penn are an inspiration. David Lynch in particular, who has the biggest Friedreich's ataxia clinic in the world, is amazing in the way he can deal with the patients, the researchers, the parents and the drug companies, trying to enroll people into clinical trials. It is an inspirational area to be working in. I have just had a student rotating in my laboratory and he has been so inspired by this that he is now thinking of doing his PhD on finding a treatment for Friedreich's ataxia.

## Q If you had unlimited resources, what would you want to do in this field?

One of the things that I find very frustrating is trying to get funding; it is extremely difficult. As I work in a very high tech environment, the field moves so quickly. It is tough always be the best in order to get the funding to keep up with everyone else. I can give you an example: one of the mass spectrometers that I have been lusting after is at least ten-times more sensitive than the best one I have now, which means the difference between working with 10 ml of blood as opposed to 1 ml of blood – that is a huge difference when you are dealing with a pediatric population. Fortunately, it looks as though we will be able to obtain one in the fall of 2015, which will be a conceptual leap for my laboratory. If I had all the resources in the world, I would have six or seven of these modern mass spectrometers working away because then I would then only be limited by my own imagination and not by my resources. You can never have too many mass spectrometers, like shoes! The other thing that I would have is some of the brightest and smartest students to come and work with me to solve some of these problems – that would be my fantasy, but it is not going to happen anytime soon!

## Q Are there any other challenges that you have come across in your work?

I think one of the challenges is that as biomarker researchers, we are very dependent on the serum samples we are given. I can give you an example: from a large number mesothelioma serum samples we actually identified a specific biomarker in every single sample that was not present in individuals exposed to asbestos that did not have mesothelioma. Then, after a tremendous amount of work, Clementina Mesaros and I figured out that the biomarker was actually lidocaine, a local anesthetic drug. What had happened was that when the patients presented at the hospital, they were given lidocaine before they had a tissue biopsy. Then after that, a blood sample was taken. As I said in one of my talks, we had discovered a wonderful biomarker of a visit a hospital! It took a significant amount of work and a lot of energy figuring out what it was. Fortunately, we did not publish our findings or we would have been very embarrassed! In essence, we are only as good as the samples we get. I think that is why it is so important to have collaborators who you can really trust and work with on a daily basis. This could be particularly pertinent in the future as we develop assays for the protein frataxin as a biomarker of treatment for Friedreich's ataxia.

## Q As you have mentioned, your laboratory uses a lot of technology such as MS. With technology typically advancing quickly, how do you think that is going to change over the next 10 years?

This is our future and we are just going to get better. What we live and die by is the resolution of the MS technology because there are so many molecules that have identical molecular weights and they might differ only in 0.001 of a Da in mass. I always think that the way to win the rat race is to invest in a better rat! At the moment, the biggest and the best commercially available mass spectrometer is working at about 500,000 resolution and it will be wonderful when they get to a million resolution – then my job will become much easier. Who could have foreseen this future? When I started using mass spectrometers, we were working with a resolution of only 1000. What used to take us a month now takes us a day. Hopefully in the future it will take us an hour – then I think I will be ready to hang my laboratory coat up. Although, I do not think I will ever actually be ready to do that!

## Q You mentioned earlier that you are the director of various centers. Can you tell us more about those roles & what they involve?

The Penn Superfund Research and Training Program Center has really consumed much of what I used to do in the Center for Cancer Pharmacology. We have six projects and five cores in the Superfund Center. Ted Emmett (Director of Community Engagement Core) has spent a lot of time going to Community Advisory Group meetings Ambler, PA where the superfund site is located, and talk about our research. We like to think of it as being bidirectional; the community comes to Community Engagement Core with problems and we try and solve them. Another important Core in my Center is the Research Translational Core, which is educating the public and various other stakeholders such as the US Environmental Protection Agency on what we do. As part of this initiative, I have been going to Washington, DC, and meeting with the congressional and senatorial staffers and telling them what we're doing, hoping that we can help them to convince their Representatives and Senators to continue funding the National Institute of Environmental Health Sciences so that the Superfund program can continue. We have Biostatistics Research Core, which helps us all with planning our experiments, and then there is an Interdisciplinary Training Core for both predoctoral and postdoctoral trainees that is run by Trevor Penning. The Superfund program is unusual because everyone who works on the individual projects is a trainee in the program. We have two Projects involving investigators from the Departments of Biology and Earth and Environmental Science in the School of Arts and Sciences, a project involving a Sociologist who has appointments in Anthropology and the Department of Family Medicine and three heavy-duty biomedical projects. We do research that goes from remediating asbestos to looking at asbestos transport, to a sociology study where we are trying to figure out why there is a cluster of mesotheliomas amongst women in Ambler, PA, USA. Is this cluster because the women washed their husbands’ asbestos-containing clothes, or for another reason? We have a project looking at the genetics of mesothelioma, because not everyone exposed to asbestos gets mesothelioma – is there a predisposition? Then we have a chemoprevention project to determine if constituents' present flaxseed can prevent mesothelioma. My project is involved in looking for biomarkers of asbestos exposure and mesothelioma.

My overall job is to ensure that this all works and we can keep funding coming in. We have another 2 years before we can go back to see whether we can get another 5 years of funding. I am also responsible for preparing the progress reports, which is a weighty job. One of the things the National Institute of Health Sciences is hot on at the moment is for us to try and commercialize some of the work that we are doing at the moment. If we come up with a remediation strategy, can we get a company to actually implement it in the field? With my biomarker work, can we patent the biomarker and use it as a blood test for mesothelioma? I have been working closely with the University of Pennsylvania Center for Innovation to find interested entrepreneurs who might want to develop this concept commercially. I also talk to the press and community groups to tell them what we are doing, so that keeps me quite busy.

In terms of my role directing the Proteomics Core, the Core is changing rapidly into a Translational Biomarker Core. This will enable researchers at Penn to analyze small molecules and lipids as well as proteins. My job in the Core is to develop new assays and present them to the Penn community. We have also successfully competed to be part of a Basic Science Center of Excellence here at Penn in the Abramson Cancer Center. The Center is focused on cancer metabolism and it uses many of the techniques that we have developed in our Translational Biomarker Core. I hope that this new center will eventually lead to the development of a Cancer Metabolism Core for the entire Abramson Cancer Center at Penn.

## Q How difficult is it to get a biomarker discovered in the first place?

It is like looking for a straw needle in a haystack; it is worse than looking for a needle that is made of stainless steel. You need the most sophisticated technology and creative researchers. We have a wonderful group here and if anyone can do it, we can!

## Q What has been your favorite moment in your career to date?

I published a paper in *Science* in 2001 demonstrating that vitamin C can induce the formation of genotoxins. I was on the west coast of Scotland at the time, where I was attending a wedding of a colleague and it was absolutely unbelievable; I walked into a newsagents shop in Campbeltown on the west coast of Scotland (where we were staying) and there were huge banner headlines of our paper with pictures of Geri Halliwell, who had apparently been taking vitamin C injections. I was standing there by the newspaper stand and I said to the guy next to me "Hey, that is our study!", and he probably thought I was a lunatic. There was so much media frenzy about it that they had to put three switchboard operators on overnight in the hotel. That week, I gave some 80 interviews to various radio and news stations and call-in shows and I was doing interviews in the car with my wife driving. Eventually, she was critiquing my interview style as we were driving along! It was the most astonishing times I have ever experienced in my life. The *Science* paper is now one of my most heavily cited papers and it gave awareness on dosing yourself up with chemicals that have not been fully studied. It was an amazing time and I still have a collection of all the headlines.

## Q Do you find it a bit hit-and-miss how things are taken by the public?

Obviously the people who make vitamin C pills were not very happy with me as there was a big drop in vitamin C sales because of me! I have a wonderful picture of a pharmacy near my laboratory saying vitamin C half price - perhaps because of my study. The media want you to make certain headline grabbing statements and they do not always fully realize the serious implications of the research that is being reported.

## Q I suppose that your current work including high community involvement would help such issues?

It does – and speaking to the general public certainly does serve to focus the mind. I remember when I was giving a talk a couple of months ago at a community meeting on asbestos, this guy stood up and started screaming at me shouting ‘where were you? Where have you been? Why did it take you so long to get here?’ I was thinking do not shoot the messenger; I am just here to help! He was so upset with the way they had been treated and I think he had every right to be, it is tricky for you to have to deal with that because they do not give you any training on how to deal with that in PhD school.

## Q Do you think they should?

Yes, I think they should. The Superfund program that I am running at the moment is unusual because the students get to go and meet people in the community and realize the relevance of what they are doing. What is fascinating is that the students really enjoy these interactions. I am very excited about our Superfund program and our work on Friedreich's ataxia because I believe we are making a real contribution to both of these areas. I will probably be talking at the next Friedreich's ataxia meeting and I am really hoping to have something worth reporting by then. These interactions with the community certainly keep our feet on the ground - there is no doubt about it.

